# Early coagulopathy after pediatric out-of-hospital cardiac arrest: secondary analysis of a randomized clinical trial

**DOI:** 10.1186/s12959-022-00422-x

**Published:** 2022-10-11

**Authors:** Dawei Zhou, Tong Li, Yi Lv, Dijia Wang, Rongli Zhang, Qing Lin, Chao Wang, Dong Zhao, Shuyang Fei, Wei He

**Affiliations:** Department of Critical Care Medicine, Beijing Tongren Hospital, Capital Medical University, Beijing, China

**Keywords:** Out-of-hospital cardiac arrest, Children, Coagulopathy, Prognosis

## Abstract

**Background:**

To estimate the incidence, risk factors, and impact on mortality and functional outcomes for early coagulopathy after the return of spontaneous circulation (ROSC) in pediatric out-of-hospital cardiac arrest (OHCA) patients.

**Methods:**

A post hoc analysis of the Therapeutic Hypothermia after Pediatric Cardiac Arrest Out-of-Hospital (THAPCA-OH) trial was conducted. Early coagulopathy was defined as presence of at least one of the following coagulation abnormalities upon admission: international standard ratio (INR), platelets, and age-adjusted activated partial thromboplastin time (APTT) within 6 h after OHCA and before therapeutic hypothermia initiation. The outcomes included 28-day mortality and functional prognosis. Multivariable logistic regression models were used to explore risk factors and association between early coagulopathy and outcomes.

**Results:**

Of the 227 patients included, 152 (67%) were male and the median age was 2.3 years [interquartile range (IQR), 0.7–8.6 years]. The overall 28-day mortality was 63%. The incidence of early coagulopathy was 46%. Lower age, longer duration of chest compression, lower temperature, and higher white blood cell (WBC) upon admission increased the risk of early coagulopathy. Early coagulopathy [OR, 2.20 (95% CI, 1.12–4.39), *P* = 0.023] was independently associated with 28-day mortality after adjusting for confounders.

**Conclusions:**

Early coagulopathy occurred in almost half of pediatric patients with OHCA. Lower age, longer duration of chest compression, lower temperature, and higher WBC increased the risk. The development of early coagulopathy was independently associated with increased mortality.

**Supplementary Information:**

The online version contains supplementary material available at 10.1186/s12959-022-00422-x.

## Introduction

Although pediatric out-of-hospital cardiac arrest (OHCA) is considered as a relatively uncommon event, the outcomes including mortality and long-term functional status are extraordinarily poor [1–5]. After return of spontaneous circulation (ROSC) following successful cardiopulmonary resuscitation (CPR), more attention should be paid to the management of post-cardiac arrest syndrome (PCAS), which had high mortality and morbidity [6, 7]. However, the pathophysiology and management of PCAS, especially for pediatric patients, has not been fully elucidated [8, 9].

Coagulation dysfunction is one of the pathophysiologic manifestations of critical illness [10, 11], which is also commonly found in pediatric patients with trauma or sepsis [12–14]. In adult PCAS patients after OHCA, the activation of blood coagulation had been demonstrated and were reported as an independent predictor for early mortality risk and poor functional outcomes [15–17]. However, the characteristics of early coagulopathy in pediatric OHCA patients were unclear.

In the present study, we aimed to describe the occurrence of early coagulopathy after pediatric OHCA and identify factors associated with the occurrence of early coagulopathy. In addition, the association between the early coagulopathy and outcomes including mortality and functional status was evaluated.

## Methods

### Study design and patients

The study included OHCA patients from the Therapeutic Hypothermia after Pediatric Cardiac Arrest Out-of-Hospital (THAPCA-OH) trial, which was conducted in pediatric ICUs at 38 children’s hospitals in the United States and Canada. The aim of the THAPCA-OH trial was to compare the efficacy of therapeutic hypothermia and normothermia [18]. The data were available in the Biologic Specimen and Data Repository Information Coordinating Center (https://biolincc.nhlbi.nih.gov). The institutional review board (IRB) approved the protocol and informed consent documents at each participating site. The secondary analysis of the data was exempted by the ethics committee of our hospital IRB.

All patients enrolled in the THAPCA-OH trial were included. Patients with lack of data of coagulation test and complete blood count (CBC) test at baseline were excluded.

### Clinical variables and outcomes

Demographic variables including age, sex, and preexisting medical condition [lung or airway disease, neurologic condition, gastrointestinal disorder, prenatal condition, congenital heart disease (CHD), and other medical condition] were extracted. The CBC test upon admission (within 6 h after OHCA and before initiation of therapeutic hypothermia) including hemoglobin, platelet, and white blood cell (WBC) and coagulation related parameters including prothrombin time (PT), international standard ratio (INR), and activated partial thromboplastin time (APTT) were recorded. The vital signs including temperature and systolic pressure were also collected. Hypotension upon admission was defined according to age-specific reference points (3 years or younger: < 60 mmHg, 4–6 years: < 75 mmHg, 7–12 years: < 80 mmHg, and 13–17 years: < 90 mmHg) [19]. Treatment of blood transfusion including cryoprecipitate, fresh frozen plasma (FFP), packed red blood cell (RBC), and platelet was also collected.

Cardiac arrest related characteristics included cardiac arrest witnessed and CPR administered by bystander or not. Primary etiology of cardiac arrest was categorized as cardiac, respiratory, other (i.e., neurological event, multiple organ system failure, drug overdose, and electrolyte imbalance), and unknown. The initial cardiac arrest rhythm included asystole, bradycardia, pulseless electrical activity (PEA), ventricular fibrillation (VF) or tachycardia (VT), and unknown. Duration of chest compression was categorized as ≤ 15 min, > 15 to ≤ 30 min, > 30 min, and unable to determine. Doses of epinephrine during CPR was categorized as ≤ 2, > 2 to ≤ 4, > 4, and missing category.

Coagulopathy upon admission was defined based on the presence of at least one of the following coagulation abnormalities: 1) INR > 1.4, 2) platelets < 100 *10^6/L, 3) APTT according to age-dependent normal coagulation values (Neonate: > 44.8, 1 month to 1 year: > 46.3, 2–5 years: > 43.8, 6–10 years: > 43.7, and 11–18 years: > 46.1) [20–22]. Because the study intended to investigate the effect of early coagulopathy on outcomes, baseline coagulation related parameters were drawn upon admission within 6 h after OHCA and before any blood transfusion and hypothermia intervention initiation.

The primary outcome was all-cause 28-day mortality. Secondary outcomes were 1-year mortality, functional status including pediatric cerebral performance category (PCPC) and pediatric overall performance category (POPC) when discharging from hospital, and second edition of Vineland adaptive behavior scales status (VABS-II) at 1-year (with VABS-II < 45 indicating profound disability, VABS-II 45–69 indicating moderate to severe disability, and VABS-II ≥ 70 indicating good functional status) [18].

### Statistical analysis

Continuous variables were shown as mean and standard deviation (SD) or median and interquartile range (IQR) after assessing for normality. Categorical variables were reported as numbers and percentages. The difference between patients with and without coagulopathy were compared by the chi-square test or Fisher’s exact test for categorical variables and the nonparametric Mann–Whitney U test for continuous variables. Same method was used to compare the characteristics for 28-day survivors and non-survivors. As for variables with missing data, the missing rate was reported. Patients with missing data were considered as a unique category for analyzing.

The multivariable logistic regression models were used to explore the predictors of early coagulopathy and the independent effect of coagulopathy upon admission on 28-day mortality, respectively. The covariates were selected using the univariate analysis with a significance level of 0.2. The final model was built by the stepwise backward elimination method based on likelihood ratio. The results of the univariate analysis and the final model were reported as odds ratio (ORs) with 95% confidence intervals (CIs). Potential multicollinearity between variables was assessed by the variance inflation factor (VIF) using “car” package of R software. The overall fit of the models was assessed by Hosmer–Lemeshow goodness-of-fit test.

The 28-day and 1-year survival were presented as Kaplan–Meier curves and compared with log-rank tests between patients with and without early coagulopathy, respectively. For patients with the vital status unknown at 1-year, the last date patients known to be alive were recorded.

The subgroup analysis was conducted as exploratory analysis. Age, sex, preexisting medical condition (prenatal condition, CHD, lung or airway disease, neurologic condition, and gastrointestinal disorder), bystanders witness, and bystander performed CRP were selected as the factors for subgroup analysis. The interaction effects of the factors on the relationship of early coagulopathy and 28-day mortality were respectively explored by the multivariable model.

All analyses were completed using R software (version 4.1.2, R Foundation for Statistical Computing). A two-sided *P* value of < 0.05 was considered statistically significant.

## Results

Of the 295 patients enrolled in the THAPCA-OH trial, after excluding patients without baseline coagulation parameters, 227 patients underwent analysis. For patients met eligibility criteria, the median age was 2.3 years [interquartile range (IQR), 0.7–8.6 years], and 152 (67%) were male (Table 1). About one half of (48%) the patients had preexisting medical condition, of which 46 (20%) patients had lung or airway disease and 34 (15%) had neurologic condition. Bystanders witnessed the cardiac arrest in 36% of the patients and CPR was administered by bystanders in 61%. Of note, the main primary etiology of cardiac arrest was respiratory condition (75%) and the main initial cardiac arrest rhythm was asystole (61%). Only 49 (22%) had duration of chest compressions less than or equal to 15 min. The overall 28-day mortality was 63%. The characteristics of the patients between 28-day survivors and non-survivors were displayed in Supplemental Table 1.

**Table 1:** Comparison of baseline characteristics of study patients

Variables	Total (*n* = 227)	No coagulopathy (*n* = 122)	Coagulopathy (*n* = 105)	*P* Value
Age, years	2.3 (0.7, 8.6)	4.0 (1.4, 11.9)	1.1 (0.4, 3.0)	< 0.001
Sex: male	152 (67)	78 (64)	74 (70)	0.366
Preexisting medical condition				
Lung or airway disease	46 (20)	25 (20)	21 (20)	0.998
Neurologic condition	34 (15)	22 (18)	12 (11)	0.229
Gastrointestinal disorder	27 (12)	16 (13)	11 (10)	0.684
Prenatal condition	24 (11)	12 (10)	12 (11)	0.863
Congenital heart disease	24 (11)	11 (9)	13 (12)	0.545
Other	54 (24)	29 (24)	25 (24)	0.997
None	119 (52)	60 (49)	59 (56)	0.357
Cardiac arrest witnessed	81 (36)	50 (41)	31 (30)	0.164
CPR administered by bystander	138 (61)	78 (64)	60 (57)	0.376
Primary etiology of cardiac arrest				0.169
Cardiac	21 (9)	16 (13)	5 (5)	
Respiratory	170 (75)	89 (73)	81 (77)	
Other	11 (5)	5 (4)	6 (6)	
Unknown	25 (11)	12 (10)	13 (12)	
Initial cardiac arrest rhythm				0.584
Asystole	139 (61)	74 (61)	65 (62)	
Bradycardia	11 (5)	7 (6)	4 (4)	
PEA	30 (13)	13 (11)	17 (16)	
Ventricular fibrillation or tachycardia	15 (7)	10 (8)	5 (5)	
Unknown	32 (14)	18 (15)	14 (13)	
Duration of chest compressions				< 0.001
≤ 15 min	49 (22)	35 (29)	14 (13)	
> 15 to ≤ 30 min	77 (34)	50 (41)	27 (26)	
> 30 min	91 (40)	32 (26)	59 (56)	
Unable to determine	10 (4)	5 (4)	5 (5)	
Doses of epinephrine				< 0.001
≤ 2	79 (35)	57 (47)	22 (21)	
> 2 to ≤ 4	65 (29)	36 (30)	29 (28)	
> 4	63 (28)	23 (19)	40 (38)	
Missing	20 (9)	6 (5)	14 (13)	
Temperature upon admission, ℃	35.8 (33.9, 37.3)	36.6 (35.2, 37.5)	34.7 (33.0, 36.1)	< 0.001
Hypotension upon admission	47 (21)	20 (16)	27 (26)	0.118
WBC upon admission, 10^9/L	13.6 (7.9, 21.1)	11.5 (6.9, 18.9)	15.4 (10.0, 24.0)	< 0.001
Hemoglobin upon admission, g/dL	12.4 (10.6, 13.9)	12.6 (11.2, 14.1)	12.2 (10.4, 13.4)	0.017

Data are median (interquartile range) or no. (%)

*CPR* Cardiopulmonary resuscitation, *PEA* Pulseless electrical activity, *WBC* White blood cell

**Fig. 1:**
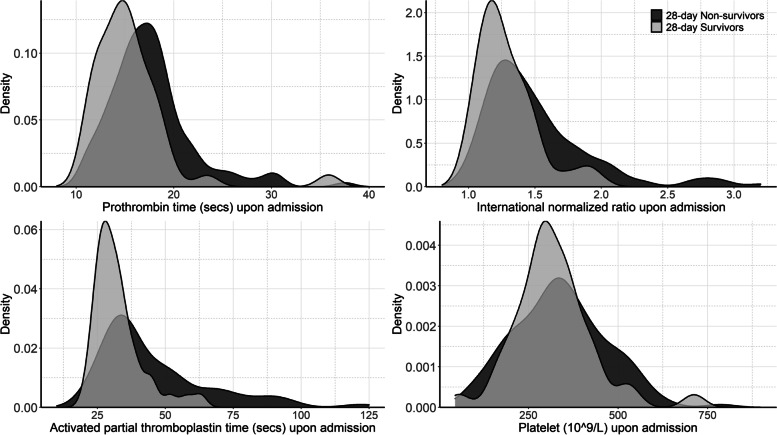
Density distribution of prothrombin time (PT), international normalized ratio (INR), activated partial thromboplastin time (APTT), and platelet count upon admission between 28-day survivors and non-survivors

According to the early coagulopathy criteria, 105 (46%) patients had cardiac arrest associated coagulopathy upon admission (Table 1). The density distribution of PT, INR, APTT, and platelet counts between 28-day survivors and non-survivors were displayed in Fig. 1. The platelet counts were similar between survivors and non-survivors; However, non-survival patients had longer PT and APTT and higher INR (Supplemental Table 1). Patients with coagulopathy were younger (1.1 VS 4.0 years, *P* < 0.001). Duration of chest compressions and doses of epinephrine used during CPR were higher in patients with coagulopathy. The admission temperature (34.7 VS 36.6 ℃, *P* < 0.001) was lower in patients with coagulopathy while WBC (15.4 VS 11.5 *10^9/L, *P* < 0.001) was higher.

**Fig. 2:**
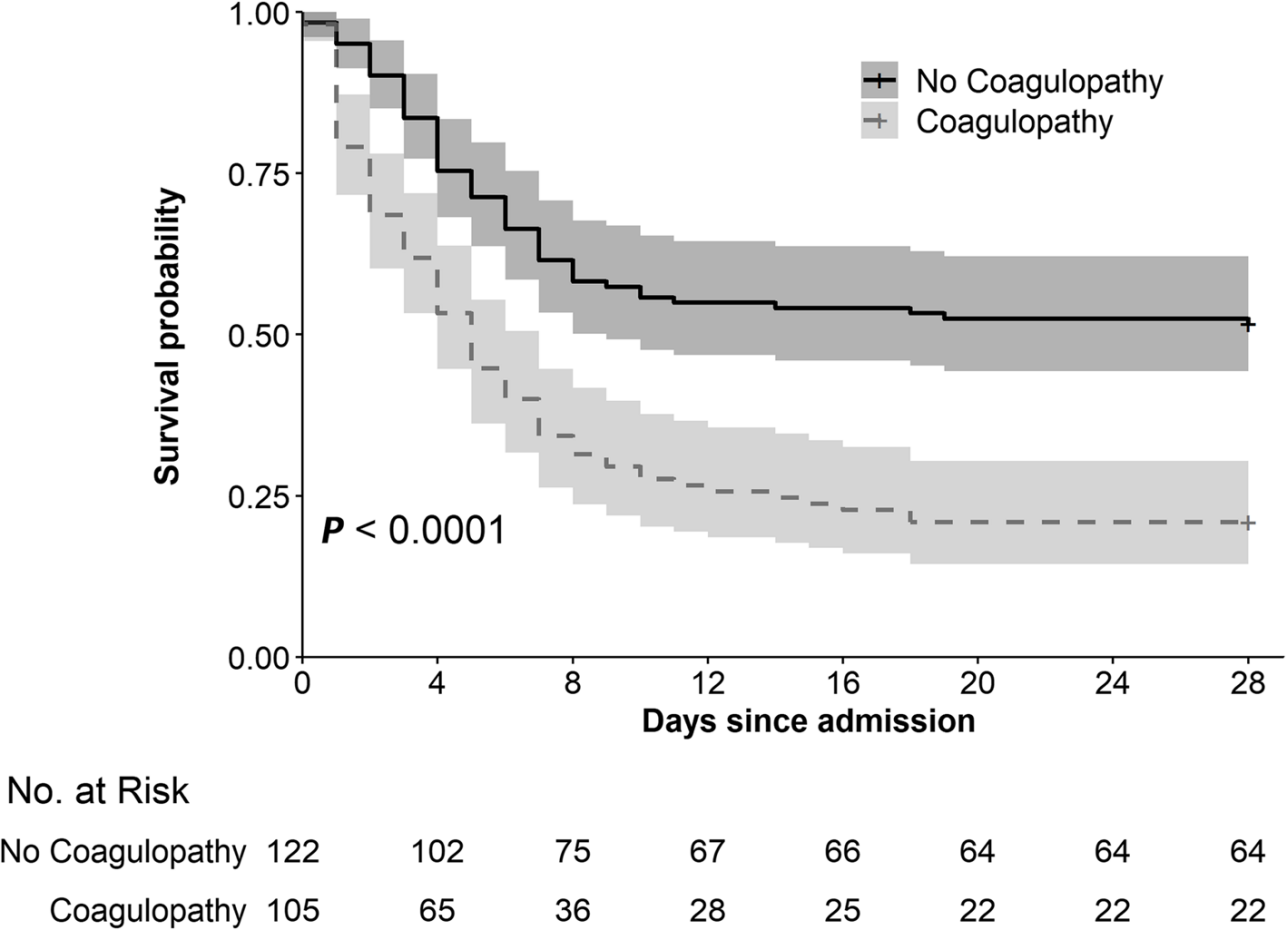
Kaplan–Meier survival curves for 28-day survival by early coagulopathy category upon admission. The difference was statistically significant according to the log-rank test (*P* < 0.001)

**Fig. 3:**
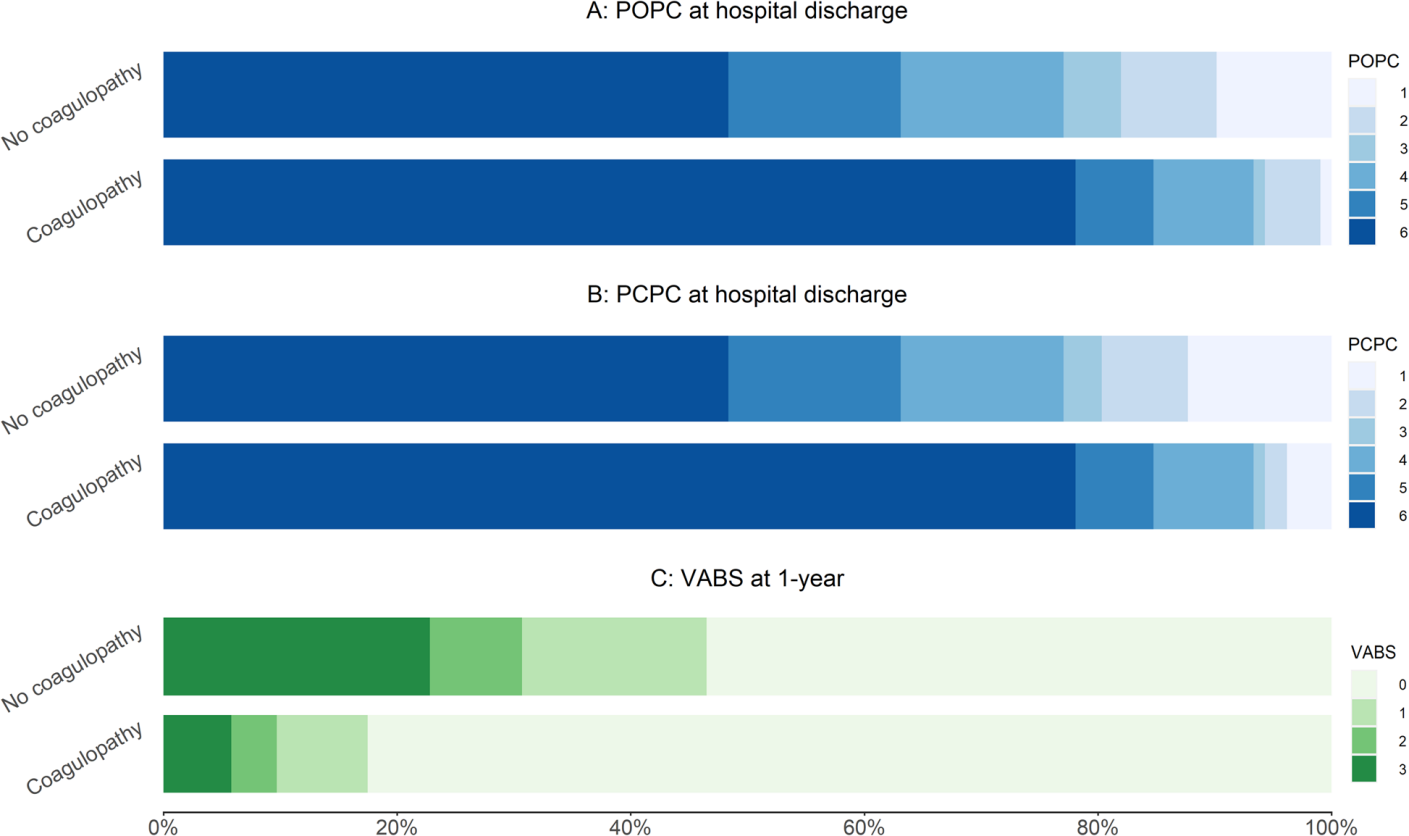
Percentage distribution of POPC at hospital discharge, PCPC at hospital discharge, and VABS at 1-year between patients with and without early coagulopathy. POPC Pediatric Overall Performance Category: 1 = Good, 2 = Mild Disability, 3 = Moderate Disability, 4 = Severe Disability, 5 = Coma or vegetative state, 6 = Death; PCPC Pediatric Cerebral Performance Category: 1 = Normal, 2 = Mild Disability, 3 = Moderate Disability, 4 = Severe Disability, 5 = Coma or vegetative state, 6 = Death; VABS Vineland Adaptive Behavior Scales: 0 = death, 1 = Profound disability (VABS < 45 or lowest possible), 2 = Moderate to severe disability (VABS 45–69), 3 = Good functional status (VABS ≥ 70)

Patients who had early coagulopathy had higher rates (79% VS 48%, *P* < 0.001) of 28-day mortality (Table 2). However, among the 142 non-survivors, the rates of causes of 28-day death were similar between the patients with and without coagulopathy. Survival over time was significantly shorter with coagulopathy than without coagulopathy for cumulative 28-day and 1-year survival (Fig. 2 and Supplemental Fig. 1, respectively; *P* < 0.001 for the comparisons of survival between the two groups by the log-rank test). Patients with coagulopathy had poor POPC, PCPC, and VABS scores (Table 2 and Fig. 3). In addition, patients alive with VABS-II score ≥ 70 at 1-year were much less in ones with coagulopathy (6% VS 23%, *P* < 0.001). It was no surprise that patients with coagulopathy received more blood transfusion, including cryoprecipitate (15% VS 1%, *P* < 0.001), FFP (49% VS 12%, *P* < 0.001), packed RBC (52% VS 31%, *P* = 0.003), and platelet (15% VS 5%, *P* = 0.016).

**Table 2:** Outcomes and blood transfusion between patients with and without coagulopathy

Variables	Total (*n* = 227)	No coagulopathy (*n* = 122)	Coagulopathy (*n* = 105)	*P* Value
**Outcomes**				
28-day death	142 (63)	59 (48)	83 (79)	< 0.001
Causes of 28-day death (n = 142)				0.602
Cardiovascular failure/futility	15 (11)	3 (5)	12 (14)	
Neurologic brain death declared	59 (42)	26 (44)	33 (40)	
Respiratory failure/futility	4 (3)	2 (3)	2 (2)	
Withdrawal for poor neurologic prognosis	57 (40)	24 (41)	33 (40)	
Withdrawal for other system failure	2 (1)	1 (2)	1 (1)	
Other	2 (1)	1 (2)	1 (1)	
Unknown	3 (2)	2 (3)	1 (1)	
POPC at hospital discharge				< 0.001
Good	13 (6)	12 (10)	1 (1)	
Mild Disability	15 (7)	10 (8)	5 (5)	
Moderate Disability	7 (3)	6 (5)	1 (1)	
Severe Disability	26 (11)	17 (14)	9 (9)	
Coma or vegetative state	25 (11)	18 (15)	7 (7)	
Death	141 (62)	59 (48)	82 (78)	
PCPC at hospital discharge				< 0.001
Normal	19 (8)	15 (12)	4 (4)	
Mild Disability	11 (5)	9 (7)	2 (2)	
Moderate Disability	5 (2)	4 (3)	1 (1)	
Severe Disability	26 (11)	17 (14)	9 (9)	
Coma or vegetative state	25 (11)	18 (15)	7 (7)	
Death	141 (62)	59 (48)	82 (78)	
Hospital length of stay	7 (3, 21)	8 (4, 28)	5 (2, 12)	< 0.001
1-year death	146 (64)	61 (50)	85 (81)	< 0.001
Alive with VABS-II score ≥ 70 at 1-year	32 (15)	26 (23)	6 (6)	< 0.001
VABS status at 1-year (n = 217)				< 0.001
Death	146 (67)	61 (54)	85 (83)	
Profound disability (VABS-II < 45)	26 (12)	18 (16)	8 (8)	
Moderate to severe disability (VABS-II 45–69)	13 (6)	9 (8)	4 (4)	
Good functional status (VABS-II ≥ 70)	32 (15)	26 (23)	6 (6)	
**Blood transfusion**				
Any blood product use	119 (53)	46 (38)	73 (70)	< 0.001
Cryoprecipitate use	17 (8)	1 (1)	16 (15)	< 0.001
FFP use	66 (29)	15 (12)	51 (49)	< 0.001
Packed RBC	92 (41)	38 (31)	54 (52)	0.003
Platelet use	22 (10)	6 (5)	16 (15)	0.016

Data are median (interquartile range) or no. (%)

*FFP* Fresh frozen plasma, *PCPC* Pediatric Cerebral Performance Category, *POPC* Pediatric Overall Performance Category, *RBC* red blood cell, *VABS-II* Vineland Adaptive Behavior Scales, second edition

After adjusted for confounders, age, duration of chest compressions, temperature upon admission, and WBC upon admission were independently associated with early coagulopathy after OHCA (Table 3). The primary etiology of cardiac arrest and hypotension upon admission were not independently associated with early coagulopathy (Table 3). Initial cardiac arrest rhythm, doses of epinephrine during CPR, temperature, hypotension, and early coagulopathy [OR, 2.20 (95% CI, 1.12–4.39), *P* = 0.023] were associated with 28-day mortality after adjusting for confounders (Table 4).

**Table 3:** Unadjusted and adjusted odds ratio for early coagulopathy after OHCA

Variables	Unadjusted ORs (95% CI)	*P* Value	Adjusted ORs (95% CI)	*P* Value
Age, years	0.88 [0.83, 0.93]	< 0.001	0.89 [0.83, 0.95]	0.001
Primary etiology of cardiac arrest				
Cardiac	1 [Reference]		1 [Reference]	
Respiratory	2.91 [1.09, 9.23]	0.046	1.55 [0.48, 5.76]	0.481
Other	3.84 [0.83, 19.54]	0.090	4.87 [0.82, 31.84]	0.086
Unknown	3.47 [1.01, 13.36]	0.056	1.76 [0.41, 8.17]	0.451
Cardiac arrest witnessed	0.58 [0.33, 1.02]	0.061	NA	
Duration of chest compression				
≤ 15 min	1 [Reference]		1 [Reference]	
> 15 to ≤ 30 min	1.35 [0.63, 2.99]	0.449	0.89 [0.37, 2.17]	0.792
> 30 min	4.61 [2.21, 10.05]	< 0.001	2.87 [1.24, 6.83]	0.015
Unable to determine	2.50 [0.61, 10.35]	0.195	1.69 [0.34, 8.33]	0.512
Doses of epinephrine				
≤ 2	1 [Reference]		NA	
> 2 to ≤ 4	2.09 [1.05, 4.21]	0.038	NA	
> 4	4.51 [2.24, 9.33]	< 0.001	NA	
Missing	6.05 [2.14, 18.97]	0.001	NA	
Temperature upon admission, ℃	0.69 [0.59, 0.79]	< 0.001	0.78 [0.66, 0.91]	0.002
Hypotension upon admission	1.77 [0.93, 3.41]	0.086	1.83 [0.81, 4.22]	0.148
WBC upon admission, 10^9/L	1.05 [1.02, 1.08]	0.002	1.04 [1.01, 1.08]	0.022
Hemoglobin upon admission, g/dL	0.89 [0.79, 0.98]	0.026	NA	

*NA* Not applicable, *OHCA* Out-of-hospital cardiac arrest, *OR* Odds ratio, *WBC* White blood cell

**Table 4:** Unadjusted and adjusted odds ratio for 28-day mortality

Variables	Unadjusted ORs (95% CI)	*P* Value	Adjusted ORs (95% CI)	*P* Value
Age, years	0.97 [0.93, 1.03]	0.322	NA	
Primary etiology of cardiac arrest				
Cardiac	1 [Reference]		NA	
Respiratory	2.98 [1.19, 7.91]	0.022	NA	
Other	1.95 [0.45, 8.96]	0.376	NA	
Unknown	4.18 [1.25, 15.25]	0.024	NA	
Cardiac arrest witnessed	0.57 [0.32, 1.00]	0.049	NA	
Duration of chest compression				
≤ 15 min	1 [Reference]		NA	
> 15 to ≤ 30 min	1.78 [0.87, 3.70]	0.119	NA	
> 30 min	4.73 [2.26, 10.22]	< 0.001	NA	
Unable to determine	2.00 [0.51, 8.68]	0.327	NA	
Initial cardiac arrest rhythm				
Asystole	1 [Reference]		1 [Reference]	
Bradycardia	0.59 [0.17, 2.16]	0.409	1.15 [0.29, 4.71]	0.841
Pulseless electrical activity	1.36 [0.58, 3.47]	0.495	1.17 [0.44, 3.30]	0.763
Ventricular fibrillation or tachycardia	0.18 [0.05, 0.56]	0.005	0.24 [0.05, 0.92]	0.046
Unknown	0.56 [0.26, 1.23]	0.145	0.55 [0.20, 1.43]	0.222
Doses of epinephrine				
≤ 2	1 [Reference]		1 [Reference]	
> 2 to ≤ 4	2.09 [1.08, 4.11]	0.03	1.52 [0.71, 3.24]	0.279
> 4	7.39 [3.39, 17.36]	< 0.001	4.04 [1.69, 10.25]	0.002
Missing	7.90 [2.41, 35.83]	0.002	6.59 [1.79, 32.36]	0.009
Temperature upon admission, ℃	0.67 [0.57, 0.78]	< 0.001	0.79 [0.66, 0.93]	0.007
Hypotension upon admission	3.09 [1.46, 7.15]	0.005	3.20 [1.34, 8.44]	0.012
WBC upon admission, 10^9/L	1.04 [1.00, 1.07]	0.028	NA	
Coagulopathy upon admission	4.03 [2.26, 7.38]	< 0.001	2.20 [1.12, 4.39]	0.023

*NA* Not applicable, *OR* Odds ratio, *WBC* White blood cell

**Fig. 4:**
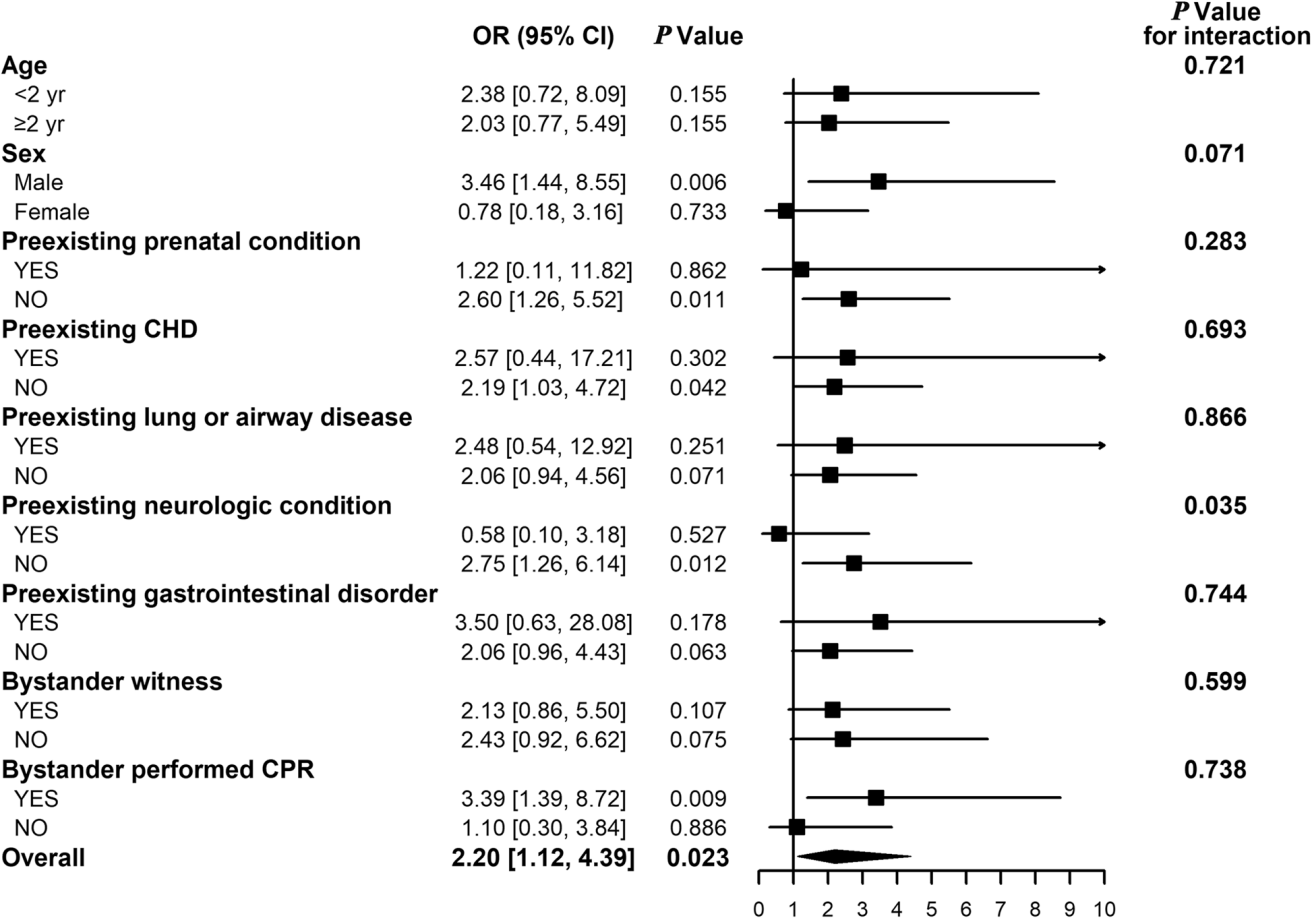
The association between early coagulopathy and 28-day mortality in different subgroup of factors including age, sex, preexisting medical condition (prenatal condition, CHD, lung or airway disease, neurologic condition, and gastrointestinal disorder), bystanders witness, and bystander performed CRP. The interaction effects of the factors on the relationship of early coagulopathy and 28-day mortality were respectively explored by the multivariable models. The covariates were selected as a priori including initial cardiac arrest rhythm and duration of chest compression due to the relatively little sample size of the event. CHD congenital heart disease, CPR cardiopulmonary resuscitation, OR odds ratio

For subgroup analysis, there was a trend that the association between early coagulopathy and 28-day mortality was obvious in male patients but not the female (Fig. 4). However, the interaction effect was not statistically different (*P* = 0.071). Interestingly, the association between coagulopathy and mortality was different in patients without preexisting neurologic condition, but not in patients with preexisting neurologic condition (*P* = 0.035 for interaction). The association between coagulopathy and mortality was not different in other prespecified subgroups.

## Discussion

In this retrospective cohort study secondarily analyzing the THAPCA-OH trial, we investigated the occurrence of early coagulopathy and the relationship between early coagulopathy and mortality outcomes after OHCA. The main findings were: 1) the early coagulopathy occurred in 46% patients after pediatric OHCA, which suggested that almost half of patients developed early coagulopathy after successful CPR; 2) lower age, longer duration of chest compression, lower temperature, and higher WBC upon admission were the major independent risk factors for early coagulopathy, while primary etiology of cardiac arrest was not found to be an independent predictor; 3) early coagulopathy was associated with higher mortality and unfavorable functional status.

The incidence of early coagulopathy after pediatric OHCA was high in the present study. These findings were consistent with the results of previous adult studies [15–17]. With the disseminated intravascular coagulation (DIC) score defined by International Society on Thrombosis and Hemostasis (ISTH), Kim et al. found that the incidence of overt DIC (DIC score ≥ 5) was 32.5% [17]. However, there were variations between adult and pediatric OHCA patients including the causes, clinical characteristics, and prognostic factors [23]. For pediatric OHCA patients, the relative studies were paramount scant. In addition, there were no gold standard criteria so far for the appropriate diagnosis of coagulopathy. Although pediatric traumatic brain injury (TBI) related coagulopathy received more attention, the diagnostic criteria of coagulopathy were also inconsistent [20, 24]. Coagulopathy was one of the common pathophysiologic conditions for pediatric critically illness [25]. In pediatric trauma patients, the incidence of early coagulopathy had been reported to range widely from 10 to 77% [12]. The incidence of sepsis-induced coagulopathy in critically ill pediatric patients even reached as high as 84.4% [13]. However, no study to date had aimed to determine the coagulopathy in the pediatric OHCA population.

The underlying mechanism of early coagulopathy after OHCA was still unknown. It was quite reasonable that patients with early coagulopathy were more frequently involved with preexisting medical conditions which could had already caused coagulopathy before cardiac arrest. However, the preexisting medical conditions were not different between patients with and without coagulopathy. Given the retrospective design, it was impossible to completely eradicate this confounding effect. Nevertheless, the early activation of blood coagulation after OHCA per se could contribute to the development of coagulopathy [15]. Previous studies showed that systemic ischemia/reperfusion, endothelial dysfunction, impaired conversion of endogenous protein C to activated protein C, and inflammatory response after OHCA could promotes coagulation activation and lead to coagulation dysfunction [15, 26]. The pathophysiology and clinical manifestations of PCAS, which was also considered as a “sepsis-like” syndrome, could produce a state similar to sepsis and contributed to multiorgan dysfunction [2, 27].

In the present study, lower age, longer duration of chest compression, lower temperature, and higher WBC upon admission were independently associated with occurrence of early coagulopathy. Previous studies suggested that most coagulation test results were dependent on age which could be attributed to developmental hemostasis [28, 29]. Plasma levels of clotting factors were significantly decreased in younger children, which could contribute to longer APTT value [28]. Even age adjusted APTT was used to define coagulopathy in this study, the reference ranges may need further validation [29]. However, whether younger patients being prone to develop coagulopathy may warrant further research. There was a significant correlation between temperature and blood coagulation, with lower temperatures leading to impaired coagulation [30]. In adult OHCA patients, early coagulopathy was associated with the degree of insult and systemic inflammation, which were coincident with our results found in pediatric OHCA [16, 17, 31].

Doses of epinephrine was a statistically significant predictor of survival, which was consistent with the study of Schindler et al. [5]. Doses of epinephrine also could reflect the duration of chest compression. Compared with other initial cardiac arrest rhythm, VF or VT was associated with survival [3, 4, 32]. Topjian et al. found early hypotension after ROSC was independently associated with lower odds of discharge survival [33]. In the present study, after adjusting for confounders of interest, early coagulopathy was an independent predictor of 28-day mortality. The role of early coagulopathy in increased 28-day mortality had not been fully elucidated. It should be prudent to infer the causal relationship between early coagulopathy and mortality. Whether early coagulopathy was just an indicator of insult from OHCA or an active trigger of following multiorgan dysfunction or both warrant further study.

For subgroup analysis, in patients with neurologic condition, the early coagulopathy was not associated with mortality, which was different from patients without neurologic condition. The finding should be interpreted with caution in the context of small sample size. In addition, the accurate diagnoses of preexisting neurologic conditions were unknown, which could influence the interpretation of the result. Interestingly, there was a trend that the relationship between early coagulopathy and mortality may be different by sex. In female patients, early coagulopathy was not associated with increased mortality. The result echoed with the finding in trauma-induced coagulopathy, which suggested female sex had a more hypercoagulable profile and conferred a survival benefit than male sex [34, 35]. However, the underlying mechanism and whether this finding was applicable to pediatric OHCA patients were unclear.

There were several obvious limitations to this study. First, the study was retrospectively designed. This study design could only show statistical association but not causality between early coagulopathy and mortality. Second, although the multivariable logistic regression models were used to adjust for potential confounders, many potential confounding factors could lead to biased results. For example, preexisting coagulopathy or several medications taken before OHCA could influence the relationship of early coagulopathy and outcomes. Third, one in five of patients were excluded due to the missing data, which could affect outcomes. Fourth, the definition of early coagulopathy in this study was based on the combination of INR, APTT, and platelet within 6 h after OHCA and before the initiation of therapeutic hypothermia. The diagnostic criteria of coagulopathy and timing of blood sample taken for the definition of “early” may need further discussion.

## Conclusions

Early coagulopathy occurred in almost half of pediatric patients after OHCA. Lower age, longer duration of chest compression, lower temperature, and higher WBC increased the risk. The development of early coagulopathy was independently associated with increased mortality.

## Supplementary Information

**Additional file 1: Supplemental Table 1.** Comparison of baseline characteristics between survivors and non-survivors. **Supplemental Figure 1.** Kaplan-Meier plots for cumulative 1-year survival according to early coagulopathy. The difference was statistically significant according to the log-rank test (*P* <0.001).

## Data Availability

The data were available in the Biologic Specimen and Data Repository Information Coordinating Center (https://biolincc.nhlbi.nih.gov).

## Abbreviations

APTT: Activated partial thromboplastin time
CBC: Complete blood count
CHD: Congenital heart disease
CI: Confidence interval
CPR: Cardiopulmonary resuscitation
DIC: Disseminated intravascular coagulation
FFP: Fresh frozen plasma
INR: International standard ratio
IQR: Interquartile range
IRB: Institutional review board
ISTH: International Society on Thrombosis and Hemostasis
OHCA: Out-of-hospital cardiac arrest
OR: Odds ratio
PCAS: Post-cardiac arrest syndrome
PCPC: Pediatric cerebral performance category
PEA: Pulseless electrical activity
POPC: Pediatric overall performance category
PT: Prothrombin time
RBC: Red blood cell
ROSC: Return of spontaneous circulation
SD: Standard deviation
TBI: Traumatic brain injury
THAPCA-OH: Therapeutic Hypothermia after Pediatric Cardiac Arrest Out-of-Hospital
VABS-II: Second edition of Vineland adaptive behavior scales
VIF: Variance inflation factor
VF: Ventricular fibrillation
VT: Ventricular tachycardia
WBC: White blood cell
