# Supplementary material for: Early coagulopathy after pediatric out-of-hospital cardiac arrest: secondary analysis of a randomized clinical trial
Source: Thromb J. 2022 Oct 11;20:62. doi: 10.1186/s12959-022-00422-x (PMC9552408; doi:10.1186/s12959-022-00422-x)
Supplement: Supplementary file 1 — Additional file 1: Supplemental Table 1. Comparison of baseline characteristics between survivors and non-survivors. Supplemental Figure 1. Kaplan-Meier plots for cumulative 1-year survival according to early coagulopathy. The difference was statistically significant according to the log-rank test (P <0.001). [file 12959_2022_422_MOESM1_ESM.docx]

**Supplementary material**

**Early coagulopathy after out-of-hospital cardiac arrest in children: secondary analysis of a randomized clinical trial**

Dawei Zhou^1^, Tong Li^1*^, Yi Lv^1^, Dijia Wang^1^, Rongli Zhang^1^, Qing Lin^1^,

Chao Wang^1^, Dong Zhao^1^, Shuyang Fei^1^, Wei He^1^

1: Department of Critical Care Medicine, Beijing Tongren Hospital, Capital Medical University, Beijing, China

*: Department of Critical Care Medicine, Beijing Tongren Hospital, Capital Medical University, Beijing, China, E-mail: tricumd@126.com

Dawei Zhou and Tong Li contributed equally.

Supplemental table 1 Comparison of baseline characteristics between survivors and non-survivors

| Variables | Total  (n = 227) | Survivors  (n = 85) | Non-survivors  (n = 142) | *P* value |
| --- | --- | --- | --- | --- |
| Age, years | 2.3 (0.7, 8.6) | 3.1 (0.9, 8.7) | 1.7 (0.5, 8.2) | 0.085 |
| Sex: male | 152 (67) | 60 (71) | 92 (65) | 0.451 |
| Preexisting medical condition |  |  |  |  |
| Lung or airway disease | 46 (20) | 15 (18) | 31 (22) | 0.556 |
| Neurologic condition | 34 (15) | 14 (16) | 20 (14) | 0.768 |
| Gastrointestinal disorder | 27 (12) | 9 (11) | 18 (13) | 0.796 |
| Prenatal condition | 24 (11) | 11 (13) | 13 (9) | 0.501 |
| Congenital heart disease | 24 (11) | 12 (14) | 12 (8) | 0.262 |
| Other | 54 (24) | 26 (31) | 28 (20) | 0.089 |
| None | 119 (52) | 40 (47) | 79 (56) | 0.265 |
| Cardiac arrest witnessed | 81 (36) | 38 (45) | 43 (30) | 0.061 |
| CPR administered by bystander | 138 (61) | 51 (60) | 87 (61) | 0.879 |
| Primary etiology of cardiac arrest |  |  |  | 0.078 |
| Cardiac | 21 (9) | 13 (15) | 8 (6) |  |
| Respiratory | 170 (75) | 60 (71) | 110 (77) |  |
| Other | 11 (5) | 5 (6) | 6 (4) |  |
| Unknown | 25 (11) | 7 (8) | 18 (13) |  |
| Initial cardiac arrest rhythm |  |  |  | 0.018 |
| Asystole | 139 (61) | 46 (54) | 93 (65) |  |
| Bradycardia | 11 (5) | 5 (6) | 6 (4) |  |
| PEA | 30 (13) | 8 (9) | 22 (15) |  |
| Ventricular fibrillation or tachycardia | 15 (7) | 11 (13) | 4 (3) |  |
| Unknown | 32 (14) | 15 (18) | 17 (12) |  |
| Duration of chest compression |  |  |  | <0.001 |
| ≤15 min | 49 (22) | 28 (33) | 21 (15) |  |
| >15 to ≤30 min | 77 (34) | 33 (39) | 44 (31) |  |
| >30 min | 91 (40) | 20 (24) | 71 (50) |  |
| Unable to determine | 10 (4) | 4 (5) | 6 (4) |  |
| Doses of epinephrine |  |  |  | <0.001 |
| ≤2 | 79 (35) | 46 (54) | 33 (23) |  |
| >2 to ≤4 | 65 (29) | 26 (31) | 39 (27) |  |
| >4 | 63 (28) | 10 (12) | 53 (37) |  |
| Missing | 20 (9) | 3 (4) | 17 (12) |  |
| Temperature upon admission, ℃ | 35.8 (33.9, 37.3) | 36.8 (35.8, 37.9) | 35.1 (33.2, 36.6) | <0.001 |
| Hypotension upon admission | 47 (21) | 9 (11) | 38 (27) | 0.006 |
| WBC upon admission, 10^9/L | 13.6 (7.9, 21.1) | 11.7 (7.0, 19.0) | 14.9 (9.0, 24.0) | 0.022 |
| Hemoglobin upon admission, g/dL | 12.4 (10.6, 13.9) | 12.2 (10.9, 13.5) | 12.5 (10.6, 13.9) | 0.613 |
| Platelet upon admission, 10^9/L | 325 (253, 405) | 302 (259, 366) | 335 (250, 421) | 0.186 |
| PT upon admission, sec | 16.2 (14.0, 18.5) | 15.0 (13.0, 16.9) | 17.2 (15.2, 19.2) | <0.001 |
| INR upon admission | 1.3 (1.2, 1.6) | 1.2 (1.1, 1.4) | 1.4 (1.2, 1.7) | <0.001 |
| APTT upon admission, sec | 35.0 (29.0, 50.5) | 31.0 (26.9, 36.0) | 39.0 (32.9, 58.5) | <0.001 |
| Hypothermia received | 118 (52) | 45 (53) | 73 (51) | 0.931 |

Data are median (interquartile range) or no. (%).

APTT activated partial thromboplastin time, CPR cardiopulmonary resuscitation, INR international standard ratio, PEA pulseless electrical activity, PT thromboplastin time, WBC white blood cell.


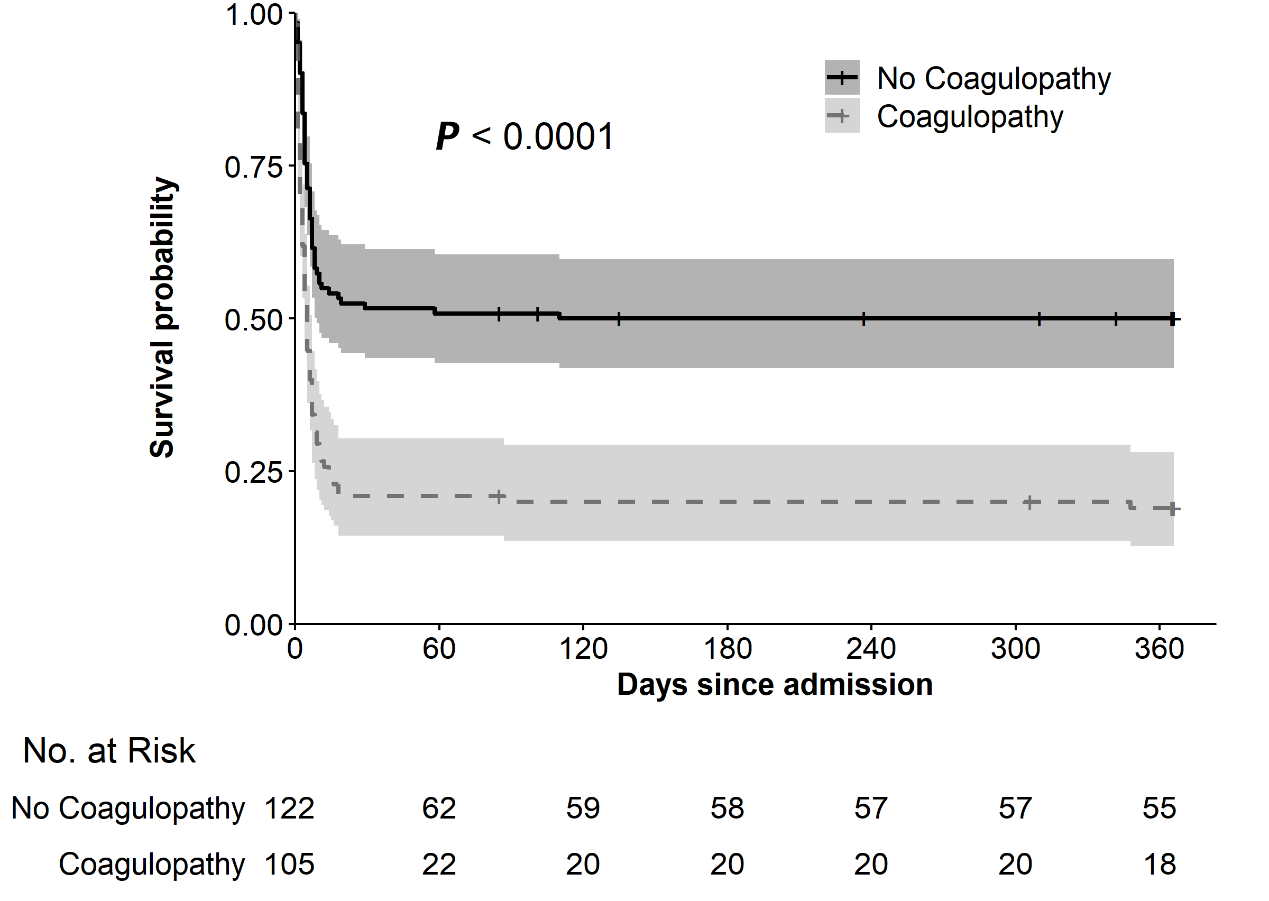


Supplemental figure 1 Kaplan-Meier plots for cumulative 1-year survival according to early coagulopathy. The difference was statistically significant according to the log-rank test (P <0.001).
